# Size-Resolved Redox Activity and Cytotoxicity of Water-Soluble Urban Atmospheric Particulate Matter: Assessing Contributions from Chemical Components

**DOI:** 10.3390/toxics11010059

**Published:** 2023-01-07

**Authors:** Athanasios Besis, Maria Pia Romano, Eleni Serafeim, Anna Avgenikou, Athanasios Kouras, Maria Giulia Lionetto, Maria Rachele Guascito, Anna Rita De Bartolomeo, Maria Elena Giordano, Annarosa Mangone, Daniele Contini, Constantini Samara

**Affiliations:** 1 Environmental Pollution Control Laboratory, Department of Chemistry, Aristotle University of Thessaloniki, GR-54124 Thessaloniki, Greece; 2 Department of Mathematics and Physics, University of Salento, 73100 Lecce, Italy; 3 Department of Biological and Environmental Sciences and Technologies, University of Salento, 73100 Lecce, Italy; 4 Institute of Atmospheric Sciences and Climate (CNR-ISAC), 73100 Lecce, Italy; 5 Department of Chemistry, University of Bari Aldo Moro, 70124 Bari, Italy

**Keywords:** WSOC, HULIS, water soluble elements, DTT assay, MTT assay

## Abstract

Throughout the cold and the warm periods of 2020, chemical and toxicological characterization of the water-soluble fraction of size segregated particulate matter (PM) (<0.49, 0.49–0.95, 0.95–1.5, 1.5–3.0, 3.0–7.2 and >7.2 μm) was conducted in the urban agglomeration of Thessaloniki, northern Greece. Chemical analysis of the water-soluble PM fraction included water-soluble organic carbon (WSOC), humic-like substances (HULIS), and trace elements (V, Cr, Mn, Fe, Ni, Cu, Zn, As, Cd and Pb). The bulk (sum of all size fractions) concentrations of HULIS were 2.5 ± 0.5 and 1.2 ± 0.3 μg m^−3^, for the cold and warm sampling periods, respectively with highest values in the <0.49 μm particle size fraction. The total HULIS-C/WSOC ratio ranged from 17 to 26% for all sampling periods, confirming that HULIS are a significant part of WSOC. The most abundant water-soluble metals were Fe, Zn, Cu, and Mn. The oxidative PM activity was measured abiotically using the dithiothreitol (DTT) assay. In vitro cytotoxic responses were investigated using mitochondrial dehydrogenase (MTT). A significant positive correlation was found between OP_m_^DTT^, WSOC, HULIS and the MTT cytotoxicity of PM. Multiple Linear Regression (MLR) showed a good relationship between OP_M_^DTT^, HULIS and Cu.

## 1. Introduction

Ambient particulate matter (PM), particularly fine particulate matter (PM_2.5_), is associated with severe short-term and long-term health effects. Although the toxicological mechanisms underlying health effects related to PM exposure are not completely understood, the generation of reactive oxygen species (ROS) and, in turn, oxidative stress are considered principal actors for the outcome of PM-related adverse health effects, particularly at the respiratory tract level. Some ROS are catalytically produced on the surface of redox-active PM [1]. Nevertheless, the majority of ROS can be generated by target cells such as alveolar epithelial cells and pulmonary macrophages upon interaction with and/or uptake of PM [2]. In turn, macrophages and dendritic cells, through proinflammatory cytokine release, can recruit other immune cells including neutrophils which are highly proficient producers of ROS during the inflammatory response [3]. Therefore, defense responses activated against PM exposure may result in a complex cascade of events that can contribute to the oxidative stress related health effects induced by PM exposure. The oxidative potential (OP) of PM (i.e., the ability of particles to generate ROS) incorporates a number of biologically relevant properties such as chemical composition, surface and particle size. Compared to PM mass alone, OP offers a more health-based exposure measure and may be a better indicator of the biologically active dose that causes adverse health effects [4]. Different OP assays capture different redox reactions that lead to the generation of different ROS species. The most commonly acellular OP measurement procedure is the dithiothreitol (DTT) assay applied either manually or online [5,6,7,8,9,10,11,12]. When considering PM exposure and the resulting health effects, the term OP^DTT^ typically signifies the chemical reactivity and possible toxicity of PM constituents related to their oxidative properties [13]. 

PM components identified as DTT-active are quinones, which are redox cycling, Humic-Like Substances (HULIS), and dissolved transition metals, mainly copper and manganese [14,15,16,17,18,19,20]. Which other PM components are utilized in the DTT assay is still a question. Furthermore, the relative contribution of PM components to the measured OP has not been thoroughly explained due to the complicated nature of PM and possible interactions between PM compounds [21]. 

In this study, size-resolved PM were collected in a typical urban site during the cold and warm periods of the year, and the OP of the water-soluble fractions were measured by the acellular dithiothreitol (DTT) assay. OP^DTT^ was further correlated with the size-resolved concentrations of various PM components including WSOC, HULIS, water-soluble elements, as well as particulate PAHs and their oxy- and nitro-derivatives (OPAHs and NPAHs). Multi linear regressions (MLRs) were performed to identify the most significant species contributing to the measured OP^DTT^. Finally, OP^DTT^ was correlated to cytotoxicity responses obtained in vitro by applying the mitochondrial dehydrogenase (MTT) bioassay in human lung cells. 

The purpose of this study was:

1. Τo gain a better understanding of the size distribution of the OP of urban PM.
2. Τo investigate its linkage to redox-active PM components.
3. To assess the relationship to cytotoxicity responses measured by cellular assays.

## 2. Materials and Methods

### 2.1. Site and Sampling Description

The site of the sampling was a major road in an urban section of Thessaloniki, the second-largest city in Greece (40°62 N/22°95 E). The sampler was placed on the roof (approximately 3.0 m above the ground) of a Central Macedonia Region air quality monitoring station. Eight (8) 48 h samplings were conducted in 2020, during the cold period (10 February–28 February 2020; n = 4) and warm period (12 May–27 July 2020; n = 4), yielding a total of forty eight (48) size-segregated samples using a five-stage high-volume cascade impactor (effective cut-off diameters of 7.2, 3.0, 1.5, 0.95, and 0.49 μm) and prebaked quartz filters, as previously described in Besis et al. (2022) [22] (Supplementary Material (SM) provides a thorough description of the sampling method). The air quality monitoring station provided the prevailing meteorological conditions (wind speed/direction, temperature and relative humidity) during sampling (Table S1). The total mass of PM collected (i.e., the sum of all size fractions) represents Total Suspended Particles (TSP).

### 2.2. Chemical Analyses

#### 2.2.1. Determination of WSOC, HULIS and Water-Soluble Elements

Extraction of ½ the slotted filters and ¼ of the backup filters after cutting was performed in 30 mL high purity MilliQ water for approximately 30 min in an ultrasonic bath and extracts were filtered through 0.22 μm PVDF membrane filters (Millipore, Burlington, MA, USA) to remove any insoluble suspended particles. The remaining parts of the filters were used to determine the concentrations of PAHs, OPAHs and NPAHs in PM along with their bioaccessibile fractions (the results were presented in our previous study [22]). For the determination of HULIS, a part of the extract was used, while the rest was reserved for the determination of other parameters such as Water-Soluble Organic Carbon (WSOC) and water-soluble metals.

The WSOC was measured in a Total Organic Carbon (TOC) Analyzer (Shimadzu, model VCSH) using the non-purgeable organic carbon (NPOC) protocol. 

For HULIS analysis, initially, the extracts were acidified to pH = 2 using hydrochloric acid. For the isolation of HULIS from the rest of the water-soluble organic compounds, the method of solid phase extraction (SPE) with appropriate columns (HLB Oasis, Waters, packing mass 200 mg, Waters) was used. It should be noted that at this pH value (pH = 2), organic compounds with acidic functional groups in their molecule (phenols, aromatic acids, aliphatic mono-di- and tricarboxylic acids, etc.) are protonated, resulting in retention of them on the HLB columns. The procedure initially involves cleaning and activating the HLB column using methanol and high-purity trace metal grade 0.01 M hydrochloric acid. After activation, the columns were dried and the samples were loaded. The exposed columns were rinsed with MilliQ water to remove the inorganic species. The SPE cartridges were dried with a nitrogen gas stream for approximately 30 min. The retained organics were eluted with methanol containing 2% (*v*/*v*) ammonia and evaporated to dryness with a gentle nitrogen stream. The flow rate was set low (~1 mL min^−1^) for the whole SPE procedure. The extracts were re-dissolved in 20 mL of MilliQ water and the concentration of HULIS-C (i.e., the carbon mass of HULIS) was determined within less than 24 h from the isolation procedure. The remaining aliquots of the HULIS fractions were kept refrigerated at ~4 °C (typically, for less than two weeks) until further analysis. The HULIS-C was also measured in a Total Organic Carbon (TOC) Analyzer (Shimadzu, model VCSH; NPOC protocol).

Water-soluble element (V, Cr, Mn, Fe, Ni, Cu, Zn, As, Cd and Pb) analyses were carried out using ICP-MS (Thermo Scientific™ iCAP™ Q). To avoid hydrolysis, the filtered aqueous extracts of PM were acidified by adding a small volume of high-purity trace metal grade HCl-HNO_3_ (2 % *w*/*v* final solution). The acidified aqueous extracts are pumped with a peristaltic pump (40 rpm) through an automatic sampler and injected into the plasma compartment with a pneumatic nebulizer (Ar 1.04 L min^−1^) through a quartz spray chamber (5 °C) and a quartz concentric torch (2.5 mm). Plasma is generated from an inert gas (argon) via a 1550 W radio frequency produced by a silver coated copper coil. At the interface region, the nickel sampling and skimmer cones operate at low vacuum (1–2 mbar). The quadrupole mass spectrometer analyzer has a resolution of 1 amu and operates at a high vacuum (10^−6^–10^−7^ mbar) generated by a turbomolecular pump. All instrument parameters are entered and controlled by appropriate software (Qtegra ISDS software). The instrument is calibrated with the external standards method and checked with appropriate CRMs. For the preparation of reagents and standards, freshly produced ultrapure water (resistivity 18.2 MΩ·cm at 25 °C) was used from a Millipore Simplicity^®^ water purification system (Millipore, Burlington, MA, USA). Trace metal grade Nitric acid (69% *w*/*w*, Thermo Fisher Scientific, Waltham, MA, USA) was used for all dilutions. Multi-element stock solution containing As, Cd, Cr, Cu, Mn, Ni, Pb, V, Zn (10 mg L^−1^, Ref N: MSE194.10.2N.L1) and single-element stock solutions of Fe (100 mg L^−1^, Ref N: M319.2NP.L1), Mn (100 mg L^−1^, Ref N: M333.2NP.L1), Zn (100 mg L^−1^, Ref N: M469.2NP.L1) all in matrix 2% HNO_3_ were obtained from CPA Chem. Stock solutions were used to prepare a series of ICP-MS calibration standards in a concentration range of 1–1000 μg L^−1^ for Fe, 1–500 μg L^−1^ for Mn and Zn and 0.05–100 μg L^−1^ for all other elements. The quality of the ICP-MS results were tested by analyzing two different certified reference materials (ERM-CA615 from the Institute for Reference Materials and Measurements EC-JRC, Belgium and SPS-SW1 Batch 128 from Spectrapure Standards Oslo Norway). The relative difference between observed mean values (for 5 measurements) and the certified reference values were found between 2.2% for Ni and 5.1% for Mn. The RSD (for 5 measurements) were ranged between 3.2% for Pb and 4.6% for Fe. Frequent tuning and calibration of the mass spectrometer ensures the proper operation of the detector. All measurements were performed using instrumental software. 

#### 2.2.2. DTT Assay

In the DTT test [4], DTT (100 μM) was added to 3.5 mL of each extracted sample of known PM mass and incubated at 37 °C in potassium phosphate buffer 0.1 M at pH 7.4 (final volume 5 mL) at different reaction time variables from 5 to 90 min. To stop the reaction at specific times, seven 0.5 mL aliquots of the mixture were mixed with 0.5 mL of 10% trichloroacetic acid. Subsequently, the seven reaction mixtures were mixed with 2 mL of 0.4 M Tris–HCl, pH 8.9 containing 20 mM EDTA, and 25 μL of 10 mM 5,5′-dithio-bis-[2-nitrobenzoic acid]. The concentration of the reaction product 5-mercapto-2-nitrobenzoic acid in each mixture was obtained by measuring the absorbance at 412 nm with a Cary 50 UV–Vis spectrophotometer (Varian Inc.). The rate of DTT depletion was obtained from the slope and the intercept of the linear regression obtained by reporting the absorbance against time, following the approach of Cho et al. [4]. The DTT depletion was normalized by the mass of the PM to obtain the intrinsic oxidative potential of the particulate matter, reported in units of pmol min^−1^ μg^−1^ (OPm^DTT^). The extrinsic oxidative potential of PM (OPv^DTT^ in units of pmol min^−1^ m^−3^), was obtained by the measured OPm^DTT^ multiplied by the total mass concentration of PM. The uncertainty on OP^DTT^ was evaluated by performing replicates on specific samples and it was, on average, approximately 10%. During all tests, care has been taken to protect DTT and DTNB reactants from light because of their instability. All OP data reported have been corrected by subtracting the DTT activity measured on field blanks.

#### 2.2.3. 3-(4,5-Dimethylthiazol-2-yl)-2,5-diphenyl Tetrazolium Bromide (MTT) Test

Cell viability of A549 cells exposed for 24 h to the PM aqueous extracts was measured by MTT assay in accordance with Latronico et al. (2018) [23]. The MTT test assesses the activity of mitochondrial dehydrogenase enzymes which catalyze the formation of formazan crystals from MTT (3-(4,5-dimethylthiazol-2-yl)-2,5 diphenyl tetrazolium bromide) in vital cells. The concentration of formazan is directly proportional to the number of viable cells. A549 cells, cultured in DMEM (Dulbecco’s Modified Eagle Medium) supplemented with 10% fetal bovine serum, 2 mM L-glutamine, 100 IU mL^−1^ penicillin, and 100 µg mL^−1^ streptomycin, were plated at a density of 1.5 × 10^4^ cells per cm^2^ into a 96-well plate. Subsequently they were exposed to the aqueous extracts (120 µL for each well) for 24 h. The osmolarity of the extracts was adjusted to 290 mOsm by adding an appropriate volume of 10 × PBS. Cells exposed to PBS represented the negative control, while cells treated with 10% DMSO represented the positive control. After incubation, 20 µL MTT (0.5 mg mL^−1^ in PBS) solution was added to each well. After incubation (4 h at 37 °C), the medium was discharged, 100 µL DMSO was added to each well and the absorbance was measured at 570 nm with a multiplate reader (EON, BioTek Instruments, Winooski, VT, USA). The inhibition of cell viability, which is indicative of cytotoxic effects, was calculated as a percentage considering the net effect of PM with respect to field blanks.

### 2.3. Quality Assurance/Control

Calibration of TOC was done with a reference curve constructed from standard potassium phthalate (KHP) solutions. The limit of detection (LOD) was calculated as three times the standard deviation (SD) of the lowest standard after five tests and was found to be equal to 0.07 mg L^−1^, while the repeatability as the ratio of the SD to the mean value was found to be 0.5%. The HULIS isolation method recovery calculation was performed using the International Humic Substances Society’s Suwannee River Fulvic Acid (1R101F) standard, which is the most widely used representative substance for atmospheric HULIS. For this purpose, solutions containing known amounts of the representative substance at different concentrations (2, 5, 10, 30 mg L^−1^) were prepared, which were subjected to the SPE procedure. The recovery of HULIS was calculated from the percentage difference of the amount of carbon before and after the SPE process. The tests performed showed recoveries greater than 80%. For each sampling campaign one slotted and one backup filter were used as field blanks. In addition, all the above procedures were also performed for the field blank filters and all concentration values presented in this work are blank corrected. 

For water-soluble elements all concentration values presented in this work were also blank corrected. The limits of detection (LODs) were calculated as three times the standard deviation (SD) of water-soluble elements’ concentrations in field blank filters. The LOD of target elements ranged between 0.02–0.05 μg L^−1^ for Pb, Ni, Cu, V, and up to 4.0 μg L^−1^ for Fe. The NIST 1648a Urban Particulate Matter standard was employed to determine the accuracy of the measurements. The treatment of the standard substance was the same as that of the samples. For the calculation of the recoveries, four samples of standard substance were used. Recoveries ranged between 82% for copper (Cu) and 120% for manganese (Mn).

### 2.4. Statistical Analysis

Microsoft Excel 2007 and SPSS version 20.0 (IBM) were used to compute all statistics. To determine statistically significant differences, the Mann–Whitney Test was employed. To investigate the relationship between OP^DTT^ and chemical compounds identified in PM, Spearman’s rank correlation coefficients and multi linear regression (MLR) were performed to detect the most important species contributing to the measured OP^DTT^ and statistical significance level was set at *p* < 0.05. In statistical analysis, data for concentration values below LOD were treated as equal to LOD/2.

## 3. Results

### 3.1. Size Distribution of PM

The mean concentration (±SD) of total collected atmospheric particulate matter (PM) mass (TSP) was 78 ± 34 μg m^−3^ in the cold period and 40 ± 19 μg m^−3^ in the warm period (*p* > 0.05), as previously described in Besis et al. (2022) [22]. Figure S1a shows the size distribution of PM. The highest mass concentrations were apparently found in particle fraction <0.49 μm, which accounted for 41% and 30% of TSP during the cold and warm periods, respectively. 

### 3.2. Size Distribution of Water-Soluble Organic Carbon (WSOC)

**Figure 1:**
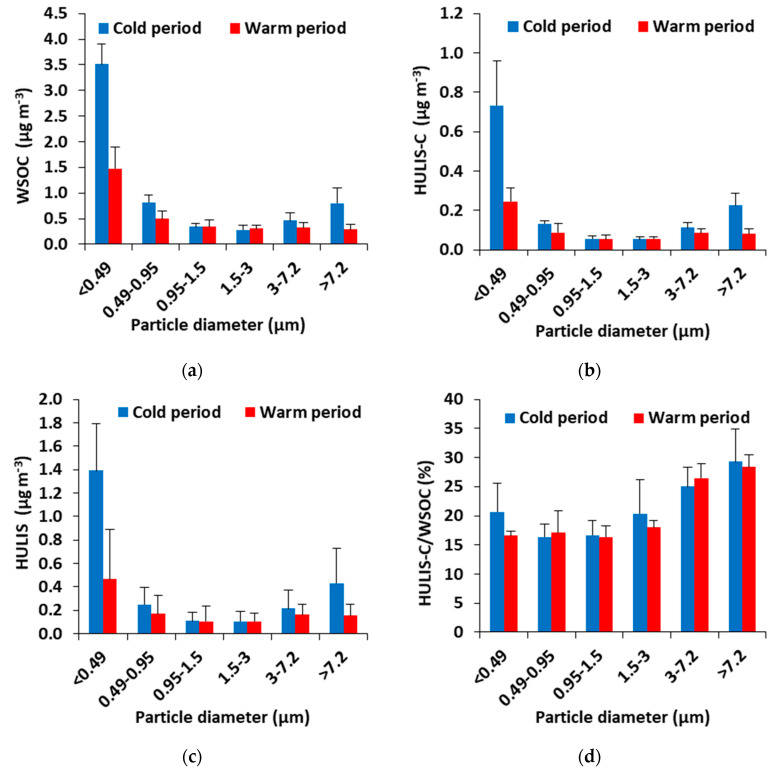
Average concentrations of: (**a**) WSOC; (**b**) HULIS-C; (**c**) HULIS; (**d**) HULIS-C/WSOC ratio; in various size fractions during cold and warm periods (Error bars show min and max).

The size-averaged concentrations of WSOC at all sampling points as well as its contribution to the particle mass are presented in Table 1. The highest total concentrations of WSOC were found in the cold period (6.2 ± 0.5 μg m^−3^), while the lowest was in the warm period (3.2 ± 0.8 μg m^−3^). WSOC values in this study are considered typical for the winter period compared to previous works [11]. The percentage contribution of WSOC to PM mass at all samples in the cold (8.8 ± 2.8%) and warm periods (9.4 ± 4.9%), is within the range of values given by literature in various parts of Europe [24,25]. An increase in the concentration of WSOC as the particle fraction decreases was observed. The highest percentages of WSOC (50–61%) were observed in the finest particle fraction (<0.49 μm), at all sampling periods, with a decreasing trend towards larger particles (12–29%; >3 μm fraction) (Figure 1a). The observed measurements are in line with the literature as most of the research shows increased concentrations in the smaller particles [24,26].

### 3.3. Size Distribution of Humic-like Substances (HULIS)

HULIS are a class of compounds that can be found in clouds, rainwater, fog, Arctic snow, and aerosols [27,28]. HULIS have attracted the interest of the scientific community because of their ability to influence the parameters of airborne particles, such as their ability to form condensation cloud nuclei (CCNs), their hygroscopicity and their optical properties [29,30]. Atmospheric HULIS consist mainly of aliphatic and aromatic organic compounds, which contain various polar functional groups (carbonyl, hydroxycarboxy and groups) [31]. Based on previous work [31], it has been shown that they show seasonal variation, mainly in urban environments, with their maximum concentrations occurring in winter and the minimum in summer. The above seasonality arises from the fluctuations of anthropogenic sources that result in their formation. Thus, combustion sources (mainly biomass) are considered their main sources during the winter, and photochemical reactions (secondary formation) during the summer. 

HULIS concentration (μg m^−3^) can be calculated from the measured concentrations of HULIS-C using a conversion factor, which is associated with the elemental composition of the HULIS fraction. The conversion factor value has been calculated in many places around the world and varies between 1.8 and 2.0 [31]. In this work, the average of the values measured in Europe (1.9) was used [24]. Table 1 includes the mean size-resolved levels of HULIS-C and HULIS. The total concentrations of HULIS were 2.5 ± 0.5 and 1.2 ± 0.3 μg m^−3^, for the two sampling periods, respectively. Concentrations determined at Thessaloniki were at the same level as those reported for various urban and suburban sites in Europe [24,32,33,34]. 

The HULIS mass distribution in the various particle fractions demonstrates the same pattern as WSOC with an increasing trend from the highest to the smallest fractions (Figure 1c). This similar distribution is to be expected, as HULIS represents a major portion of the WSOC mass [35]. At all sampling periods, the highest mass abundance of HULIS was found in the particle fraction <0.49 μm (45–63%, 34–51%, in the cold and warm periods, respectively). These findings are consistent with other studies that showed the prevalence of HULIS in the accumulation mode [24,36,37].

The total HULIS-C/WSOC ratio ranged from 17 to 26% (Figure 1d) for all sampling periods, confirming that HULIS are a significant part of WSOC. The values from this study agreed well with the values from previous studies [31,33].

### 3.4. Size Distribution of Water-Soluble Elements

**Figure 2:**
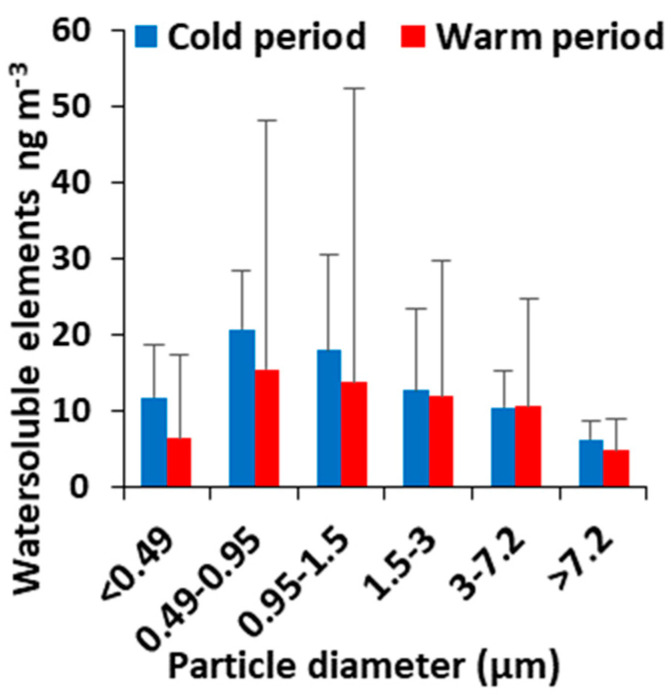
Average concentrations of water-soluble elements (cold and warm periods).

Mean bulk concentrations of water-soluble elements (V, Cr, Mn, Fe, Ni, Cu, Zn, As, Cd and Pb) for all particle fractions were 80 ± 34 ng m^−3^ (cold period) and 116 ± 114 ng m^−3^ (warm period) (Table S2). The most abundant water-soluble elements were Fe, Zn, Cu, and Mn with bulk concentrations averaging 48 ± 15, 15 ± 11, 7.8 ± 4.0 and 3.7 ± 2.2 ng m^−3^, respectively for the cold period and 62 ± 60, 27 ± 35, 14 ± 12 and 5.7 ± 6.1 ng m^−3^, respectively for the warm period. On the contrary, V, Pb, Cr, Ni, As and Cd were found at significantly lower concentrations. Metals exhibited variable mass size distributions (Figure 2) depending on their origin (fuel combustion, vehicle exhaust emissions, tire wear and brake, road dust resuspension) and chemical form. Ni, As, Mn and Cr peaked at <0.49 μm exhibiting a clear decreasing trend with the increase in particle size. Fe, Cd, Pb and V peaked at 0.49–0.95 μm, while Cu, Zn at 0.95–1.5 μm. Transition metals can be significant contributors to OP^DTT^ of ambient PM. It has been reported that transition metals (i.e., Cu (II) and Mn(III)) attributed ~80% of DTT consumption by ambient PM, while the remaining 20% was contributed by quinones and other redox-active species. Experiments with pure metals showed that their DTT-activity follow the order Cu(II) > Mn(II) > Co(II) > V(V) ~ V(III) ~ Ni(II) > Pb(II) ~ Fe(II) > Fe(III) [15].

### 3.5. Size Distribution of PAHs, OPAHs and NPAHs

Concentrations of PAHs, OPAHs and NPAHs in PM were presented in our previous study along with their bioaccessibility [22]. In brief, the mean total particulate concentrations of ∑_16_PAHs, ∑_6_NPAHs and ∑_8_OPAHs were 18 ± 6.7, 0.37 ± 0.11 and 0.94 ± 0.29 ng m^−3^, respectively, in the cold period sampling campaign, and 6.4 ± 2.1, 0.25 ± 0.08 and 0.09 ± 0.01 ng m^−3^, respectively, in the warm period (Table S3). Significant seasonal variances were observed only for PAHs and OPAHs (*p* < 0.05). The largest portion of total particulate levels of ∑_16_PAHs, ∑_6_NPAHs and ∑_8_OPAHs were related to particles <0.49 μm (Figure S1b–d).

### 3.6. DTT Redox Activity of Size-Resolved PM

**Figure 3:**
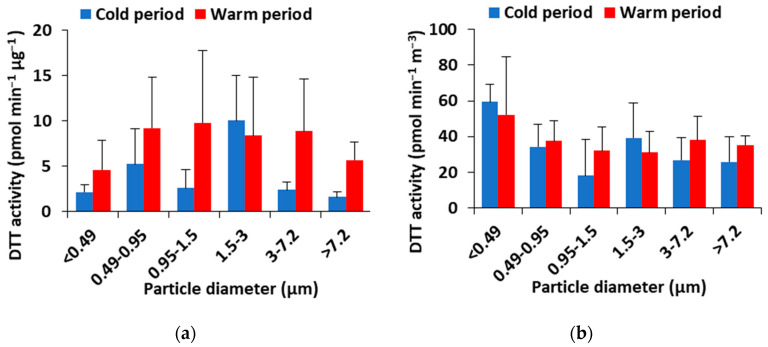
Average concentrations of: (**a**) OPm^DTT^; (**b**) OP_V_^DTT^ in various size fractions during the cold and warm periods (Error bars show min and max).

The mass-normalized OP (OP_m_^DTT^; pmol min^−1^ μg^−1^) and the volumetric OP (OP_v_^DTT^; pmol min^−1^ m^−3^) activity calculated in the different particle size fractions during the two sampling periods are shown in Figure 3. While the mass-normalized OP is an indicator of the intrinsic toxicity caused by PM, related to its source, the OP_v_^DTT^ is thought to be an appropriate measure for assessing inhalation exposure to redox-active PM [38].

The average OP_m_^DTT^ measured in the different particle size factions was in the cold period 24 ± 13 pmol min^−1^ μg^−1^ and significantly higher in the warm period (47 ± 31 pmol min^−1^ μg^−1^). This seasonal pattern was observed in all size fractions with the exception of some particle fractions (1.5–3 μm) that showed increased intrinsic DTT activity in winter rather than in summer. Elevated OP^DTT^ levels in the cold period are primarily due to the fact that organic aerosol formation is supported in winter due to the increasing primary emissions (biomass burning or coal), and due to enhanced partitioning of redox-active semi-volatile organic compounds (SVOCs) in the particulate phase [39,40,41]. On the other hand, elevated OP^DTT^ levels in summer are usually attributed to enhanced formation of secondary organic aerosols (SOAs) that are rich in oxidized organic compounds [42], as well as due to enhanced fresh traffic emissions and/or traffic-induced road dust resuspension [11]. 

OPm^DTT^ values found in this study in all PM size fractions (8.7–84 pmol min^−1^ μg^−1^) were significantly lower than those previously reported in related studies from ground-level sites in Thessaloniki (urban background site: 120–460 and 30–340 pmol min^−1^ μg^−1^ vs. heavy-traffic site: 20–310 and 50–270 pmol min^−1^ μg^−1^ in the cold and the warm period, respectively [11]). The size-specific distribution data revealed that the lowest OP_m_^DTT^ occurred in the quasi-ultrafine fraction (<0.49 µm) which means that this fraction contained less water-soluble DTT-active compounds than larger diameter particles [43]. Results from literature studies performed on size-dependent OP are variable. Velali et al. (2016) [11], found the maximum OP_m_^DTT^ at 0.49–0.95 μm, in Thessaloniki, although larger particles in the accumulation mode 0.97–3 μm also exhibited remarkable DTT activity. Shafer et al. (2016) [7], found that the median OP_m_^DTT^ of PM from roadside motorway, roadside canyon, and background urban sites in six European cities (including Thessaloniki) followed the order PM_3–7_ (13.5 nmol min^−1^ mg^−1^) > PM_3_ (9.5 nmol min^−1^ mg^−1^) > PM_>7_ (<5 nmol min^−1^ mg^−1^). However, the majority of studies reported that the highest intrinsic OP was exhibited by submicrometer fine PM. Fang et al. (2017) [6], found unimodal distribution for water-soluble OPmDTT peaking in quasi-ultrafine particles (0.1–0.18 μm). Similarly, Lyu et al. (2018) [18] reported a bimodal distribution for OPm^DTT^ with the major peak between quasi-UF mode and accumulation mode (0.1–0.32 μm). 

The average OP_V_^DTT^ in the different particle fractions was 204 ± 89 pmol min^−1^ m^−3^ and 226 ± 88 pmol min^−1^ m^−3^ for the cold and warm periods, respectively (*p* > 0.05). For OPv^DTT^, a clear peak in the <0.49 mm size fraction was obvious (Figure 3) suggesting that inhalation exposure to redox-active PM and the associated health risk are highest for these particles that reach the deepest parts of the human respiratory tract.

### 3.7. MTT Cytotoxicity of Size-Resolved PM

The cytotoxic potential of the water-soluble fraction of size segregated airborne particles was assessed by the MTT test on A549 cells that were exposed to the extracts for 24 h. The results are shown in Figure S2. During the winter period, six samples showed an inhibition of cell vitality above 20%, in any case below 50%, while in the summer period five samples showed an inhibition of cell vitality higher than 20% with two samples showing an inhibition of about 60 and 50%, respectively. Moreover, some samples, in particular during the summer period, showed hormesis, represented by negative value of the % inhibition. Hormesis consists of a biphasic dose–response to an environmental agent with a low dose stimulation and a high dose inhibition of a biological function [44]. The stimulatory effect of low concentrations of toxic chemicals has been already described by previous authors in A549 cells [45].

### 3.8. Relationships between OP^DTT^ and Chemical Compounds

Relationships between OP^DTT^ and chemical components were examined by correlation analysis and MLR analysis. Correlation analysis is usually employed to identify the specific PM compounds as contributors to DTT activity since, although they do not show causation, they may indicate the emission sources (as source tracers, e.g., levoglucosan, HULIS and K^+^) [1].

The Spearman’s rho correlation coefficients calculated for OP_m_^DTT^ and OP_v_^DTT^ with the corresponding mass and air volume normalized concentrations of chemical compounds of PM are shown in Table S4. A significant negative correlation (−0.70; *p* < 0.01) was found between OP_m_^DTT^ and the mass concentration of PM, in agreement with other studies [46,47,48], with a result that certain species that contribute to total PM mass do not contribute significantly to ROS generation capability [46]. No correlation was found with OP_v_^DTT^ and PM mass appeared to moderately correlate with OP_m_^DTT^ (0.41; *p* < 0.01), nevertheless, no correlation was found with the PM mass concentration suggesting that OP, related to the composition of PM rather than their mass. 

In our study a significant positive correlation (*p* < 0.01) was found between OP_m_^DTT^ and WSOC and HULIS in agreement with previous studies [6,11,16,38,39]. OP_m_^DTT^ exhibited positive correlations, with Cr, Mn, Fe, Cu and Pb (*p* < 0.01). Also, a significant correlation was found for OP_v_^DTT^ with the volumetric concentrations of Cr (0.30; *p* < 0.05). Other studies also reported strong correlations of OP_v_^DTT^ with metals [39,49]. No correlations were observed between OP_m_^DTT^/OP_v_^DTT^ and organic compounds.

The non-biological dithiothreitol (DTT) assay provides a good measure of the oxidative potential of PM as a surrogate for the initial step in biological induction of oxidative stress [5]. Numerous in vitro studies have demonstrated a direct correlation between the chemical oxidative potential of PM estimated by DTT consumption and its ability to induce cellular oxidative stress responses [50,51]. Although the relationships between OP measurements and acellular assays and the effects at the cellular level, as well as the health effects are not fully understood [52], studies have shown that the OP^DTT^ of PM is directly correlated to its ability to induce cellular oxidative stress responses [11,51,53]. In this study, a significant correlation (0.31; *p* < 0.05) was observed between OP_m_^DTT^ and the MTT cytotoxicity of PM indicating that similar components likely affect PM’s ability to form reactive oxygen species (ROS), which are accountable for decreased mitochondrial functions, cell damage, and death [11]. Cytotoxicity also has significant correlations with WSOC, Cu, Mn, Cr, Fe, As, Pb and Cd (Table S4). 

To obtain further insight into the dominant PM constituents linked with OP measurement, studies have employed multiple linear regression analysis (MLR) [54]. The MLR analysis performed in this study yielded a good relationship (R^2^ = 0.84) between OP_m_^DTT^, HULIS (*p* < 0.01) and Cu (*p* < 0.01) as predictors. The coefficient of each independent variable shown in Equation (1) represents the intrinsic water-soluble DTT activity of the specific PM species.

OP_m_^DTT^ (pmol min^−1^μg^−1^) = (16.2 ± 5.5) × HULIS (pg μg^−1^) + (0.9 ± 0.1) × Cu (pg μg^−1^) (1)

## 4. Discussion

Dithiothreitol-based oxidative potential (OP^DTT^) was measured and evaluated in relation to the chemical makeup of PM (<0.49, 0.49–0.95, 0.95–1.5, 1.5–3.0, 3.0–7.2 and >7.2 μm) collected at an urban traffic site in the city of Thessaloniki, in North Greece with a focus on WSOC, HULIS and water-soluble elements (PAHs, OPAHs and NPAHs results were reported in our previous study [22]). WSOC and HULIS, dominated the water-soluble PM fractions with strong dependence upon size range. The most abundant water-soluble elements were Fe, Zn, Cu, and Mn. Metals exhibited variable mass size distributions. OP^DTT^ of ambient PM may largely vary depending on particle size fraction and contributing sources. Mass size distribution of OP_m_^DTT^ of PM was almost the same as water-soluble elements. The MTT cytotoxicity of PM peaked in the 0.49–0.97 μm size range in the cold period and in the 3–7.2 μm size range in the warm period.

Multiple Linear Regression (MLR) study showed a good relationship between OP_M_^DTT^ and HULIS and Cu. A significant correlation was observed between OP_m_^DTT^ and the MTT cytotoxicity of PM indicating that similar components likely affect PM’s ability to form reactive oxygen species (ROS), which are accountable for decreased mitochondrial functions, cell damage, and death. 

The present study highlights the need for further research to reasonably identify and apportion the contribution of chemical components and emission sources to the observed OP of ambient PM. This study may have limitations in terms of sample size and seasonal representability, which could be addressed in future research. The sample size (48 size-segregated samples) was likely insufficient to provide complete information about seasonal variations in the concentrations of the targeted compounds. Future studies will attempt more comprehensive PM composition characterization, accounting for interactions between PM components, and a greater number of OP measurements.

## 5. Conclusions

WSOC and HULIS dominated the water-soluble PM fractions. The highest percentages of WSOC and HULIS were observed in the submicron size fractions, which typically have long residence time in the atmosphere. The total HULIS-C/WSOC ratio confirms that HULIS are a significant part of WSOC. The most abundant water-soluble elements were Fe, Zn, Cu, and Mn. Metals exhibited variable mass size distributions depending on their origin. 

A significant negative correlation was found between OP_m_^DTT^ and the mass concentration of PM implying that OP is primarily an intrinsic property of PM related to its composition rather than its mass. Also, a significant positive correlation was found between OP_m_^DTT^ and WSOC—HULIS and the MTT cytotoxicity of PM. A Multiple Linear Regression (MLR) model was investigated using OP_m_^DTT^ as dependent variables indicating HULIS as the major predictor. Further research is essential to better understand the toxic impacts of PM composition.

## Figures and Tables

**Table 1:** Mean (±standard deviation) size-resolved concentrations of PM mass, WSOC, HULIS-C and HULIS in the cold and warm sampling periods.

Particle Diameter (μm)	PM (μg m^−3^)		WSOC (μg m^−3^)		HULIS-C (μg m^−3^)		HULIS (μg m^−3^)	
	Cold Period	Warm Period	Cold Period	Warm Period	Cold Period	Warm Period	Cold Period	Warm Period
<0.49	31.8 ± 15	11.8 ± 3	3.51 ± 0.41	1.48 ± 0.42	0.73 ± 0.23	0.24 ± 0.07	1.39 ± 0.43	0.47 ± 0.13
0.49–0.95	8.7 ± 4.3	5.1 ± 3.2	0.81 ± 0.14	0.5 ± 0.16	0.13 ± 0.02	0.09 ± 0.04	0.25 ± 0.03	0.17 ± 0.08
0.95–1.5	5.9 ± 3.4	5 ± 3.4	0.34 ± 0.07	0.34 ± 0.13	0.06 ± 0.02	0.05 ± 0.02	0.11 ± 0.03	0.1 ± 0.04
1.5–3	4.6 ± 2.4	4.9 ± 2.4	0.28 ± 0.09	0.31 ± 0.07	0.05 ± 0.01	0.05 ± 0.01	0.1 ± 0.03	0.1 ± 0.02
3–7.2	11.1 ± 4.2	6.1 ± 3.9	0.46 ± 0.15	0.32 ± 0.09	0.11 ± 0.03	0.08 ± 0.02	0.21 ± 0.05	0.16 ± 0.04
>7.2	15.6 ± 3.8	6.8 ± 2.6	0.8 ± 0.3	0.29 ± 0.1	0.23 ± 0.06	0.08 ± 0.02	0.43 ± 0.12	0.15 ± 0.05
Total	77.7 ± 33.5	39.5 ± 18.5	6.2 ± 0.52	3.23 ± 0.76	1.31 ± 0.25	0.61 ± 0.15	2.5 ± 0.48	1.15 ± 0.28

PM: Particulate Matter; WSOC: water-soluble organic carbon; HULIS: humic-like substances; HULIS-C: carbon mass of HULIS. Cold period: 10 February 2020–28 February 2020 (n = 4); Warm period: 12 May 2020–27 July 2020 (n = 4).

## Data Availability

Not applicable.

## Supplementary Materials

The following supporting information can be downloaded at: https://www.mdpi.com/article/10.3390/toxics11010059/s1, Table S1: Summary of sampling information; Table S2: Mean (±standard deviation) size-resolved concentrations (ng m^−3^) of water-soluble elements in the cold and warm sampling periods; Table S3: Summery statistics of total* PAH, NPAH, OPAH concentrations for the cold and warm periods (ng m^−3^); Table S4: Spearman’s rho correlation coefficients of OPmDTT, OPvDTT and MTT cytotoxicity (MTT-reduction mg^−1^ PM), with the corresponding mass and air volume normalized concentrations of chemical compounds of PM; Figure S1: Average concentrations of (a) PM, (b) PAHs, (c) NPAHs and (d) OPAHs in various size fractions during the cold and warm season (Error bars show min max); Figure S2: Average concentrations of MTT-reduction activity (% of the control), in various size fractions during the cold and warm season (error bars show min and max.
