# Supplementary material for: Size-Resolved Redox Activity and Cytotoxicity of Water-Soluble Urban Atmospheric Particulate Matter: Assessing Contributions from Chemical Components
Source: Toxics. 2023 Jan 7;11(1):59. doi: 10.3390/toxics11010059 (PMC9867266; doi:10.3390/toxics11010059)
Supplement: Supplementary file 1 [file toxics-11-00059-s001.zip › toxics-2112216-supplementary.pdf]

Size-Resolved Redox Activity and Cytotoxicity of Water-Soluble Urban Atmospheric Particulate Matter: Assessing Contributions from Chemical Components

Athanasios Besis [1*], Maria Pia Romano [2], Eleni Serafeim [1], Anna Avgenikou [1], Athanasios Kouras [1],
Maria Giulia Lionetto [3], Maria Rachele Guascito [3,4], Anna Rita De Bartolomeo [3],
Maria Elena Giordano [3], Annarosa Mangone [5], Daniele Contini [4] and Constantini Samara [1]

[1] Environmental Pollution Control Laboratory, Department of Chemistry, Aristotle University of Thessaloniki, GR-54124, Thessaloniki, Greece
[2] Dipartimento di Matematica e Fisica, Universita del Salento, 73100, Lecce, Italy
[3] Dipartimento di Scienze e Tecnologie Biologiche e Ambientali, Universita del Salento, 73100, Lecce, Italyc
[4] Istituto di Scienze dell'Atmosfera e del Clima, ISAC-CNR, 73100, Lecce, Italy
[5] Dipartimento di Chimica, Universita degli Studi di Bari Aldo Moro, 70124, Bari, Italy

* Correspondence: Athanasios Besis; athanasb@chem.auth.gr; Tel.:+30-231-099-7858

Sampling

Quartz filters (Environmental Tisch TE-230QZ) were used as impaction substrates (slotted 5.7 x 5.7 cm) for the five coarser size fractions and rectangular backup filters (2500QATUP) for the finest size fraction. Preceding sampling, filters were cleaned by "baking" at 450 °C for 5 h, wrapped in aluminum foil and sealed in polyethylene zip bags. Unloaded and loaded filters were weighed in a KERN 870 semi-microbalance (resolution 10 μg) after 48 h equilibration at relative humidity 50±5% and temperature 20±0.5 °C. Loaded filters were stored in the dark, wrapped in aluminium foils and sealed in polyethylene zip bags, at -20 °C until extraction and analysis.
Sampling was carried out using a 5-stage high-volume cascade impactor (Sierra instruments, model 235) operated at a constant flow rate of 1.1 m^3 min^{-1}. Effective cut off diameters were: 7.2, 3.0, 1.5, 0.95 and 0.49 μm for the 5 impaction stages, respectively. No substantial drop (<5%) in flow rate was observed at the end of each sampling. The sampling period was 48 hours and the sampling volume was approximately 3250 m^3.

Table S1. Summary of sampling information

			Meteorological data			
	**A/A**	**sampling date**	Temperature (^{O}C)	RH (%)	Wind speed (m s^{-1})	Wind directio n
**Cold season**	1	10/2-12/2/2020	15	71	0.72	S
	2	19/2-21/2/2020	14	63	2.11	N
	3	24/2-26/2/2020	19	55	1.70	SSW
	4	26/2-28/2/2020	15	46	2.94	SE
**Warm season**	5	12/5/-14/5/2020	27	61	1.11	SSW
	6	15/5/-18/5/2020	30	61	0.86	SSW
	7	20/5/-22/5/2020	24	66	2.33	N
	8	25/7/-27/7/2020	30	63	1.51	SW

Table S2. Mean (± standard deviation) size-resolved concentrations (ng m^{-3}) of water-soluble elements in cold and warm sampling period.

	Cold period	Warm period	Cold period	Warm period	Cold period	Warm period	Cold period	Warm period	Cold period	Warm period	Cold period	Warm period
**Particle diameter (μm)**	**V**		**Cr**		**Mn**		**Fe**		**Ni**		**Cu**	
<0.49	0.09±0.05	0.16±0.19	0.81±0.38	0.84±0.18	0.87±0.72	0.87±0.7	5.76±2.35	6.29±5.1	0.12±0.06	0.19±0.2	1.31±1.02	2.02±1.65
0.49-0.95	0.13±0.1	0.08±0.05	0.6±0.53	0.45±0.43	0.78±0.45	0.64±0.67	10.36±3.21	15.41±18.33	0.11±0.04	0.14±0.17	1.83±0.58	3.06±3.24
0.95-1.5	0.09±0.01	0.07±0.08	0.3±0.45	0.65±0.44	0.71±0.56	0.88±1.17	8.27±3.98	13.71±15.03	0.09±0.04	0.08±0.08	2.17±1.26	4±4.22
1.5-3	0.11±0.05	0.09±0.1	0.24±0.4	0.77±0.27	0.57±0.64	1.06±1.12	9.48±8.1	11.78±9.6	0.07±0.02	0.08±0.06	1.3±0.81	2.75±1.77
3-7.2	0.09±0.01	0.08±0.07	0.16±0.26	0.36±0.33	0.57±0.51	1.7±2.15	8.41±3.09	10.56±9.59	0.07±0.03	0.1±0.1	0.89±0.72	1.37±0.84
>7.2	0.07±0.08	0.09±0.05	0.11±0.17	0.38±0.35	0.21±0.13	0.55±0.34	5.41±1.84	4.65±2.81	0.05±0.01	0.05±0.04	0.29±0.07	0.58±0.24
Total	0.58±0.2	0.57±0.53	2.21±1.3	3.45±0.49	3.72±2.2	5.7±6.11	47.7±14.65	62.4±59.75	0.51±0.18	0.65±0.66	7.79±3.97	13.79±11.82
	**Zn**		**As**		**Cd**		**Pb**		**Σ_{10}metals**			
<0.49	2.37±2.11	2.22±2.67	0.11±0.06	0.11±0.1	0.15±0.11	0.09±0.09	0.12±0.09	0.07±0.05	11.7±6.9	12.9±10.9		
0.49-0.95	5.79±2.35	7.08±8.93	0.09±0.03	0.1±0.08	0.32±0.17	0.28±0.29	0.68±0.29	0.48±0.57	20.7±7.8	27.7±32.8		
0.95-1.5	5.92±6.19	11.62±17.04	0.05±0.02	0.05±0.04	0.14±0.12	0.27±0.34	0.12±0.08	0.29±0.28	17.9±12.7	31.6±38.7		
1.5-3	0.8±0.72	4.71±4.94	0.02±0.01	0.03±0.01	0.022±0.013	0.093±0.085	0.02±0.03	0.09±0.03	12.6±10.8	21.5±18		
3-7.2	0.15±0.2	1.24±1.12	0.03±0.01	0.03±0.02	0.006±0.004	0.03±0.02	0.01±0.02	0.02±0.01	10.4±4.9	15.5±14.3		
>7.2	0.04±0.08	0.4±0.34	0.02±0.01	0.02±0.01	0.006±0.004	0.01±0.007	0.01±0.01	0.01±0.02	6.2±2.4	6.7±4.2		
Total	15.06±11	27.26±34.58	0.32±0.12	0.34±0.25	0.65±0.39	0.78±0.82	0.98±0.34	0.96±0.92	79.5±34.4	116±114		

Table S3: Summery statistics of total* PAH, NPAH, OPAH concentrations for the cold and warm periods (ng m^{-3}) (Besis et al., 2022).

PAHs										
	**Mean**		**Median**		**Max**		**Min**		**SD**	
Compounds	***Cold***	***Warm***	***Cold***	***Warm***	***Cold***	***Warm***	***Cold***	***Warm***	***Cold***	***Warm***
Chry	0.395	0.082	0.361	0.065	0.757	0.160	0.100	0.038	0.294	0.059
Acy	0.214	0.055	0.202	0.056	0.360	0.085	0.089	0.024	0.127	0.028
Ace	0.060	0.030	0.042	0.022	0.126	0.061	0.028	0.015	0.047	0.022
F	0.153	0.024	0.139	0.022	0.270	0.039	0.064	0.013	0.093	0.012
Ph	1.330	0.140	1.230	0.133	2.230	0.221	0.643	0.075	0.732	0.068
An	0.689	0.173	0.548	0.167	1.250	0.250	0.414	0.108	0.380	0.074
Fl	2.320	0.780	2.120	0.791	3.350	1.160	1.680	0.384	0.764	0.368
Py	2.820	0.689	2.520	0.689	4.150	1.020	2.090	0.356	0.950	0.309
B[a]An	2.800	1.150	2.330	1.140	4.740	1.620	1.810	0.715	1.340	0.409
Chry	2.770	1.080	2.410	1.090	4.320	1.660	1.940	0.513	1.070	0.475
B[b]Fl	1.760	0.693	1.440	0.624	3.010	1.080	1.160	0.446	0.873	0.288
B[k]Fl	1.560	0.801	1.320	0.626	2.750	1.490	0.863	0.459	0.875	0.473
B[a]Py	0.273	0.152	0.231	0.115	0.471	0.348	0.161	0.031	0.147	0.141
I[1,2,3-cd]Py	0.233	0.168	0.215	0.153	0.340	0.255	0.164	0.112	0.081	0.066
dB[a,h]An	0.349	0.246	0.298	0.241	0.601	0.348	0.198	0.155	0.179	0.085
B[ghi]Pe	0.215	0.134	0.173	0.083	0.377	0.343	0.135	0.028	0.114	0.144
**$\sum_{16}$PAHs**	**17.9**	**6.4**	**15.9**	**6.3**	**27.6**	**9.0**	**12.4**	**4.1**	**6.7**	**2.1**

NPAHs										
	**Mean**		**Median**		**Max**		**Min**		**SD**	
Compounds	***Cold***	***Warm***	***Cold***	***Warm***	***Cold***	***Warm***	***Cold***	***Warm***	***Cold***	***Warm***
1-NNAP	0.011	0.012	0.011	0.010	0.014	0.022	0.007	0.005	0.003	0.007
2-NNAP	0.013	0.019	0.013	0.016	0.017	0.037	0.007	0.005	0.005	0.014
5-NACE	0.033	0.014	0.034	0.013	0.040	0.023	0.023	0.006	0.007	0.008
3-NBP	0.027	0.013	0.027	0.012	0.034	0.020	0.021	0.008	0.006	0.006
4-NBP	0.099	0.049	0.089	0.045	0.160	0.081	0.057	0.024	0.046	0.025
3-NFLT	0.080	0.064	0.079	0.063	0.128	0.098	0.035	0.030	0.040	0.029
1-NPYR	0.105	0.079	0.104	0.079	0.139	0.104	0.075	0.052	0.028	0.023
**$\sum_{7}$NPAHs**	**0.368**	**0.248**	**0.353**	**0.246**	**0.500**	**0.342**	**0.264**	**0.159**	**0.105**	**0.082**

OPAHs										
	**Mean**		**Median**		**Max**		**Min**		**SD**	
Compounds	***Cold***	***Warm***	***Cold***	***Warm***	***Cold***	***Warm***	***Cold***	***Warm***	***Cold***	***Warm***
((1,4)O_2NAP)	0.043	0.004	0.046	0.004	0.071	0.007	0.007	0.000	0.028	0.003
9-OFLN	0.048	0.004	0.037	0.004	0.096	0.007	0.023	0.002	0.033	0.002
AceNQ	0.040	0.004	0.038	0.003	0.066	0.006	0.019	0.002	0.021	0.002
(9,10)O_2ANT)	0.093	0.008	0.095	0.008	0.135	0.011	0.046	0.004	0.040	0.003
2-N-9-OFLN	0.028	0.002	0.022	0.002	0.056	0.004	0.013	0.001	0.020	0.001
(O2BAA)	0.215	0.016	0.181	0.016	0.376	0.025	0.121	0.009	0.113	0.007
BaOFLN	0.139	0.010	0.121	0.009	0.216	0.015	0.099	0.006	0.054	0.004
(BAN)	0.406	0.037	0.382	0.036	0.588	0.049	0.270	0.027	0.139	0.009
**$\sum_{8}$OPAHs**	**0.940**	**0.085**	**0.863**	**0.085**	**1.330**	**0.101**	**0.698**	**0.070**	**0.285**	**0.014**

* Sum of 6 particle size fractions (<0.49, 0.49-0.95, 0.95-1.5, 1.5-3.0, 3.0-7.2 and >7.2 μm); Cold period 2020; Warm period 2020; SD: Standard Deviation

Table S4: Spearman's rho correlation coefficients of **OP_m^{DTT}, OP_v^{DTT}** and MTT cytotoxicity (MTT-reduction mg^{-1} PM), with the corresponding mass and air volume normalized concentrations of chemical compounds of PM.

**Spearman's rho correlation coefficients**	**OP_m^{DTT}**	**OP_v^{DTT}**	**MTT cytotoxicity**
PM	-0.70**		
OP_m^{DTT}		0.41**	0.31*
WSOC	0.41**		0.32*
HULIS	0.36*		
Cr	0.57**	0.30*	0.54**
Mn	0.69**		0.38*
Fe	0.56**		0.44*
Cu	0.39**		0.70**
As			0.47*
Cd			0.32*
Pb			0.28*

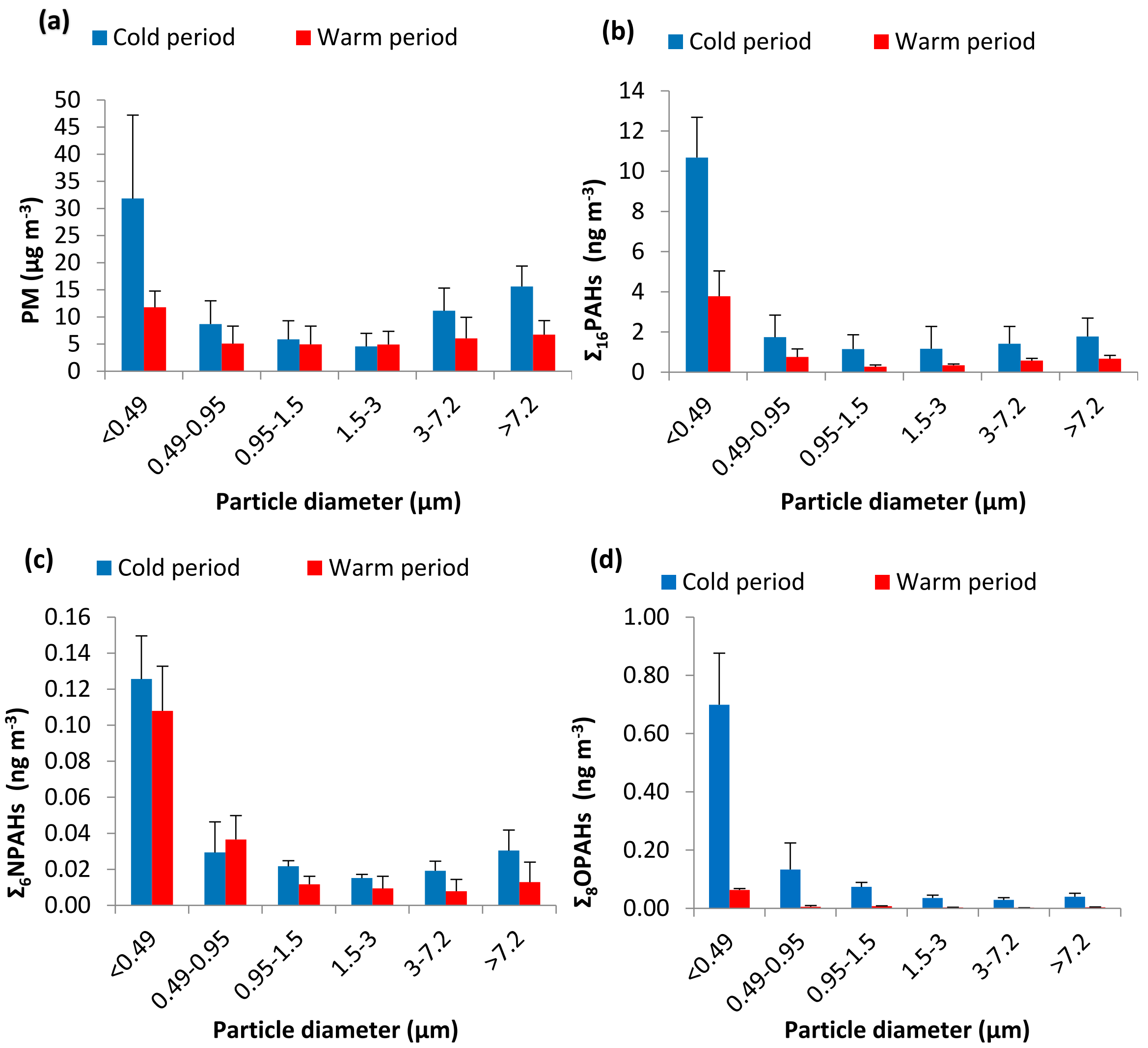

Figure S1. Average concentrations of (a) PM, (b) PAHs, (c) NPAHs and (d) OPAHs in various size fractions during cold and warm season (Error bars show min max) (Besis et al., 2022).

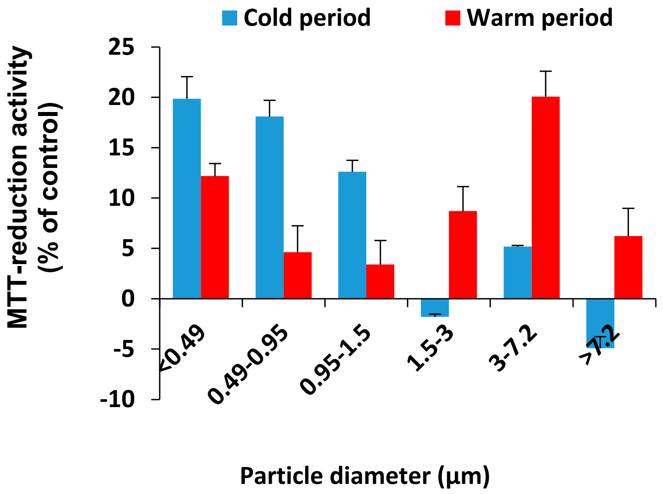

Figure S2. Average concentrations of MTT-reduction activity (% of the control), in various size fractions during cold and warm season (Error bars show min max).

)
